# PW02-031 - Genetic and clinical manifestations of CAPS

**DOI:** 10.1186/1546-0096-11-S1-A172

**Published:** 2013-11-08

**Authors:** AK Bybee, H Lachmann, E Omoyinmi, B Nedjai, P Woo, T Lane, S Savic, P Hawkins, M McDermott

**Affiliations:** 1 National Amyloidosis Centre, UCL, London, UK; 2 Infectious Diseases, Institute of Child Health, London, UK; 3 Institute of Bio-Medical Engineering, Imperial College, London, UK; 4 Immunology and Molecular Pathology, London, UK; 5 Medicine, UCL, London, UK; 6 Clinical Immunology and Allergy, London, UK; 7 Leeds Institute of Rheumatic and Musculoskeletal Medicine, University of Leeds, Leeds, UK

## Introduction

Many variants in the NLRP3 gene are associated with a particular spectrum of autoinflammatory diseases or hereditary recurrent fevers (HRFs), including familial cold autoinflammatory syndrome (FCAS), Muckle Wells Syndrome (MWS), and chronic infantile neurologic, cutaneous and articular syndrome (CINCA), also known as neonatal onset multisystem inflammatory disease (NOMID). These are now subsumed under the umbrella term CAPS (cryopyrin-associated periodic syndromes). However, CAPS is rarely encountered in any population and is often difficult to identify. Extension of genetic screening to a genomic scale may soon become routine, but that development will require definition of the identities and ontology of the constellations of symptoms involved, in order for bioinformatics applications to keep pace. Patients with clinical features suggestive of these conditions were analysed for NLRP3 variants.

## Objectives

To explore the prevalence of NLRP3 variants in patients with autoinflammatory disorders in order to establish a consistent pattern of associated symptoms.

## Methods

Genomic DNA from 91 unrelated patients referred for genetic testing for any of the NLRP3*-*associated diseases was screened for variants in exon 3 of NLRP3 by PCR and automated DNA sequencing. Diverse symptoms were recorded when possible and compared with the outcome of genetic testing.

## Results

Heterozygous missense substitutions in NLRP3 (NM_001243133.1) were found in 16 of the 91 patients (17.6%), including p.V198M, p.D303N, p.P315L, p.T348M, p.V351M, p.T436I, p.G569R, and p.Y570F variants. In addition to the well-known CAPS signs of recurrent fever, urticarial rash and sensorineural hearing loss, symptoms relatively frequently reported in NLRP3 variant-positive patients included chronic meningitis or headache (9/14 with NLPR3 variant vs. 6/38 without) uveitis or iritis (8/15 vs. 10/41), growth retardation (6/14 vs. 5/37) epiphyseal or patellar growth (5/14 vs. 1/35) and learning difficulties (4/14 vs. 3/35). In addition, several patients with multiple symptoms characteristic of CAPS were mutation-negative, and at least 9 patients with or without a genetic diagnosis developed AA amyloidosis.

## Conclusion

In addition to the more typical symptoms, this study highlighted distinctive neurological characteristics of some CAPS cases. These results indicate a large degree of genetic and symptomatic heterogeneity in NLRP3-related syndromes and suggest that other, as yet unidentified, genes and other factors may be involved in producing a CAPS-like phenotype. Such factors accrue to produce symptomatic recurrent fever disease with an accompanying heightened risk of developing AA amyloidosis. Autoinflammatory diseases continue to drive investigation of the genes controlling fundamental aspects of the innate immune system.

## Competing interests

None declared

